# Miscellanies

**Published:** 1843-01-01

**Authors:** 


### Miscellanies.

### MESMERIC AMPUTATION.

ON Tuesday, the 22d November, a great gun was fired in the Royal Medico-Chirurgical Society, which was expected to demolish the whole tribe of sceptics who have opposed the occult arts of mesmeric necromancy, for some time past. Dr. Elliotson headed the corps, which was a very numerous one—(some forty mesmeric disciples having got into the camp as visitors)—and he had generals, captains, civil engineers, barristers, chirurgeons, and others, *minoris notæ*, in his train. Of the prudence which guided the counsel in admitting the paper in question, we will say nothing. Sir B. Brodie and others condemned it; and, at the beginning, we were of the same opinion; but, before the evening was over, we were satisfied that "all was for the best." The cause was opened by Counsellor Topham, who somewhat triumphantly observed that, as the paper contained nothing but authentic and incontrovertible *facts*, he would not make a single comment, nor offer a word of explanation.

The facts of the case were shortly these. A labouring man suffered for five years from disease of the knee-joint, ending in ulceration of the cartilages. He had continued to work, though in great pain, and with increasing emaciation, till he was received into the "*district hospital at Willow, near Ollerton Notts.*"* It was not till the 9th September, that Mr. Topham saw him, and then commenced the mesmeric processes, which he continued daily, with more or less effect in setting the sufferer to sleep. Mr. Ward, the surgeon, was absent for most part of the time between the above date and the 1st or 2d October, the day of operation. Mr. Topham, the Counsellor, had the patient, therefore, all to himself, and there can be little doubt that he *counselled* him to some purpose. Be that as it may, on the 1st of October the mesmeriser, after exhibiting a *quantum suf.* of the unknown influence, threw the patient into a profound coma, and took the precaution to keep his own fingers on the patient's eyelids during the amputation, which was performed by Mr. Ward. "*Soon after the second incision, a moaning was heard from the patient, which continued at intervals until the conclusion.*" "It gave me the idea of a troubled dream." When the operation was over, the man seemed a little bewildered, and then exclaimed—"*I bless the Lord to find it's all over.*" How did he know this, if he were in a profound state of insensibility all the time? When questioned, he observed that he felt no pain, but once "*felt as if he heard a kind of crunching,*"—evidently the sawing of the bone. The man is recovering.

Mr. Ward's part of the account adds little to Counsellor Topham's, and is corroborative of the lawyer's statement. But the surgeon lets out one or two particulars of a most unfortunate nature, which, in the discussion at the Society afterwards, turned the Great Gun against the gunners themselves. Mr. Ward observes that "the extreme quivering or rapid action of the divided muscular fibres *was less than usual.*" And again, "*I touched the divided end of the sciatic nerve, without any increase of the low moaning.*" Ah! Mr. Ward, we fear you have not studied the "*reflex function*" under our friend Marshall Hall, or you would never have marked the above passage in *Italics!!* But more of that anon. What now is the amount of all these *facts,* which were to silence the Society and the profession? The whole amounted to this miserable *non sequitur*—this "lame and impotent conclusion"—that *non-expression* of pain is a proof of its *non-existence!!* Is it not humiliating to medical science that a barrister, a physician, and one or two surgeons, should come forth in a learned and royal society to uphold such a piece of ratiocination as the above in the present day. Mr. Alcock, Dr. Johnson, and others exposed its fallacy, while Sir B. Brodie and others shewed that nothing was more common than patients undergoing the most painful operations, without wincing, without saying a word, or *evincing* the slightest feeling of pain. But would any man, who had an ounce of brain, or an atom of reason, believe that these patients had no *sensation* of pain at the time? Certainly not. They felt the pain; but they had command over their feelings, and betrayed no sensation of agony at the moment.

The mesmeric party cut a remarkably poor figure, and looked not a little foolish when Dr. M. Hall rose, and said that the operation in question proved a great deal too much; for, unless the Notts farmer was constituted in a different manner from all other animals, the instant the sciatic nerve of the amputated limb was touched—even if the patient had been previously decapitated, (which must have induced a tolerable degree of coma), the other extremity, which lay motionless, would have kicked most manfully! This experiment, therefore, he asserted, proved beyond the possibility of a doubt, that the quiescence of the man, under the operation, was a voluntary effort of the will controlling the feel-

* What are the nature and extent of "Willow District Hospital?" We only know that Willow is a small *parish* of some four or five hundred inhabitants.

ings of suffering. Beaten at all points, the Leader of the forlorn hope was obliged to fall back on the mesmeric sleep, so often induced by the Barrister, and the improvement of the man's health during the time he was being daily mesmerized at the hospital. That the pokings and pawings, the strokings and clawings of the necromancer should have often set the poor ignorant peasant asleep, or apparently asleep, we do not deny—and that the rest, comfort, and other means secured by an hospital, should have produced an amended state of health, we may also admit, without subscribing to the monstrous absurdity, and the anti-physiological phenomena of the narrative in question. The party, however, notwithstanding all their mustering, manœuvering, and packing, were completely put to the rout in every direction! They may now appeal to the *gaube-mouches* of the public; but they will not soon try their mountebank tricks again before the profession.

### A MAN WITH THREE TESTICLES.

Dr. Macann, Staff-Surgeon at the recruiting depôt, Coventry, has lately met with a curious anomaly in a recruit of good stature, and well formed. There were two testes on the right side, (one above the other) and one on the left. On accurate examination the spermatic cords were all distinctly traced to the respective testicles.

### AQUA CHALYBEATA.

This preparation is the invention of Messrs. Bewley and Evans, of Dublin. In six ounces of the solution there are 13 grains of citrate of iron, dissolved in water highly charged with carbonic acid gas, and flavoured with orange peel. We have used some bottles of it, and have found it the most grateful and refreshing chalybeate we have ever swallowed. It is to be got now at the principal chemists in London.

### DR. SYMONDS' INTRODUCTORY LECTURE AT THE BRISTOL SCHOOL OF MEDICINE.

This is a very sensible and practical lecture, in which the Lecturer recommends his pupils to pursue the eclectic rather than the dogmatic or hobbyhorsical systems of practice—but that without timidity, or wavering between different systems. We have only room for a short extract on the subject of Hydropathy, or rather Hydromania.

"If we are curious to investigate the circumstances to which hydropathy owes its great popularity, it will probably be found that the following are some of the principal causes:—First, the system is very simple—diseases may be manifold, but the remedy is single, though variously applied; second, the universal applicability of the remedy seems to harmonise with its universal distribution over the face of the globe; third, the system dispenses with the use of drugs, most of which are disagreeable, and many not a little dangerous; fourth, it gives ample employment to the superabundant energies of many of the invalids who seek its aid—physicians are often at a loss in recommending interesting occupation to the hypochondriac, to whom nothing is interesting but what has reference to his health; fifth, diaphoresis or sweating, which is one of the great results of the system, is in much favor with invalids. Whether from the additional notions of bygone pathology, or from instinctive feelings (I suspect the former), patients always attach great importance to perspiration; doubtless this is really one of the most advantageous results of the process. I know a gentleman who for many years has had the greatest difficulty in procuring action of his bowels, and to him the sudorific process of the water cure has been obviously beneficial in compensating the alvine deficiencies. Lastly, the outward use of cold water allays many disagreeable sensations, and at the same time the feeling of glow or re-action gives the idea of an invigorating effect often far beyond what is really imparted. Multitudes of patients of the neurotic class find the sensations attendant upon cold water a pleasant exchange for the anomalous and often distressing feelings which haunt them when their minds are not otherwise occupied—such are dysæsthesia and dysphoria in their various forms; moreover, the use of cold water begets the desire of its repetition. Every one who has accustomed himself to plentiful ablutions of cold water, when debarred from their use feels all the annoyance consequent on the loss of a stimulus. He may fancy that what he experiences is owing to an accumulation of what ought to be removed from the skin, but the time, the lapse of a day for instance, is often too short for any such effect. The real cause I believe to be the privation of the accustomed excitement of the cutaneous nerves and vessels. This fact gives, I think, a ready clue to the explanation of the perseverance with which hydropathic patients often follow up the system—'increase of appetite hath grown by what it feeds on.'

"As I began with observing, some useful additions to practice may, no doubt, be gathered from the observation of the peculiar methods adopted by the hydriatic professors; but eventually, after the present popular excitement has passed away, cold water will, like all other remedies, find its proper level; it will be known as a valuable expedient in certain diseases, and under certain circumstances, many of which are yet to be learned. The supposed universality of its powers, as I have just remarked, is one of its chief attractions at present; and when that has been disproved, as it surely will be, there is ground for fearing that the remedy will sink in popular opinion to a degree which will be the exact measure to its present undue elevation. The dyspeptic, the hypochondriacal, the nervous, and the victims of ennui and satiety, as they at one time invoked the mysteries and mummeries of mesmerism—at another swallowed, at the bidding of an empiric, hundreds of vegetable pills—or at another, flew for aid to Wildbad and Baden-Baden, and Schlangenbad, and were disappointed—will now not unnaturally appeal to hydropathy, and perhaps with some better success. But the subjects of fevers, exanthemata, and acute visceral inflammations, the unhappy sufferers from organic diseases, and those who have been broken-down by hæmorrhages and exhausting discharges, and those who require the skilful knife of the surgeon—all, in fact, who are in direst need of the remedial art, will, we venture to say, remain adherents of the practice which has been built upon the confirmed observation of disease for successive centuries, and upon the accumulated improvements which every year had added to the knowledge of the human body in health."

---

### Hepatic Abscess—Operation.

N. A. Woods, Esq., Madras Medical Service, ætat. 44, of weakly constitution, much emaciated, and in appearance twenty years beyond his actual age. Served 21 years in India. Had been weakly and dyspeptic for some years previously to retiring, but never suffered from any acute tropical disease. Quitted Madras in April 1841, and had a severe attack of dyspepsia at Bombay the latter end of that month. Arrived in England in June 1841, in a weakly and emaciated condition; when, for the first time, so far as he knows, hepatic derangement became evident, followed very soon by enlargement of the organ. He treated himself with blue-pill and aperients; but this plan never afforded him any relief. In October the tumor extended down suddenly (he declares in one night) into the central and lateral portions of the umbilical region, but without any other sensation at the time than feeling of distention and fulness. He got through the Winter and Spring of 1841-42 but badly, constantly ailing, and using alteratives, &c. as before. In the months of April and May, and after excessive torture from the passage of gall-stones, his health improved perceptibly, as evidenced by increase of muscular power and flesh. On the 28th May, 1842, he began the use of the cold-bath, in the use of which he persisted daily till the middle of August, when a great increase in the size of the tumor, with obtuse pain, manifested themselves. On this occurrence Mr. Woods sought the advice of Dr. James Johnson, who being absent, requested Mr. Martin to visit him. The tumor was then hard and painful when pressed, and there was a distinct and separate tumor of the cæcum—the health miserably reduced—pulse 96—bowels constipated—no rigors or cold sweat—urine scanty and high-coloured. Mr. Martin prescribed the nitro-muriatic acid bath, with the regular use of purgatives, and the occasional use of the warm-water bath.

In about a month the tumor subsided considerably, (that of the cæcum being no longer perceptible,) with attendant improvement in the general health. He was therefore recommended by Dr. Johnson, who then saw him with Mr. Martin, to continue the same plan of treatment. Shortly after this last-mentioned consultation, Mr. Woods went out of town, and was seized, while there, with catarrh and diarrhœa, for which he unhappily used mercurials, though specially advised against them by both Dr. Johnson and Mr. Martin. From this time he rapidly declined in strength; and, on presenting himself on the 28th September, Mr. Martin thought he could perceive fluctuation in the tumor. The patient's emaciation was extreme—pulse 120, with night delirium—no rigors or cold sweats, either now or previously—tongue bright red, dry, and excoriated. There was much pain in the tumor, especially about its centre, indicating inflammatory action in its peritoneal surface, and a probable adhesion of it to the parietes of the abdomen:—for, there was a distinct hardness around the centre of four or five inches in extent, and alteration in the position of the body caused none in the site of fluctuation.

Under these circumstances, Mr. Martin considered that an opening into the abscess might afford a chance, how remote soever, of saving the patient's life. He therefore requested Mr. Woods to come into town with the view to his better care, and immediately consulted with Dr. Johnson and Mr. Henry James Johnson, who both agreed as to the existence of an abscess, though very deep-seated. On the 1st October Mr. Johnson explored with the needle, when a little pus was discovered by him in the groove; and on the following day he passed a common-sized trocar deep into the cavity of the abscess, when the contents were found so thick as hardly to admit of evacuation. About six ounces of flaky viscid pus did however escape, when the canula was allowed to remain carefully covered over with a large poultice. He had been taking anodynes during the last few nights, the diet being light and nourishing, with a little wine, and the bowels being regulated by the mildest means.

October 3d. Passed a better night, and is altogether easier since the operation—no delirium—pulse reduced to 90—pus discharging freely on the poultice, and the patient expressing himself as vastly relieved.

4th. Had an increase of fever last night, with dryness of the tongue, but continues to feel quite easy—secretions in full activity—no delirium. The diet carefully regulated, and the anodyne at bed-time.

5th. The canula was withdrawn last evening, notwithstanding which he had more fever than before, but it subsided towards morning, on getting rid of much discharge—pulse 96—tongue moist—feels quite easy—secretions active.

6th. Discharging freely at the opening, and feeling easy, but more weakly—secretions copious—pulse 100—no delirium.

7th. Rested better—less fever—secretions healthy and abundant—pulse 100—feels weakly.

Ordered some Indian ale in lieu of wine.

8th. Continues easy, but becoming daily more reduced in strength. Whenever the discharge is moderated or arrested he experiences much uneasiness, and sometimes actual pain in the region of the liver, but these instantly subside on a vent being given to the matter—pulse 96—secretions active as before—no delirium.

9th. Was seized at 2 A.M. with violent pain in the site of the abscess, which caused him suddenly to get out of bed. This exertion produced a copious jet from the opening, when he felt quite relieved, and obtained some rest. His strength, however, rapidly sunk from this time; and a collapse, which no stimulants could overcome, terminated his existence at noon, the patient retaining his faculties till within a few minutes of his death.

*Dissection.*—Body emaciated.

*Thorax.* Universal and firm adhesions of surfaces of both pleuræ. Structure of lungs pretty healthy. Cavity of pericardium containing about ℥j. of fluid. Heart rather larger than natural, loaded with fat. Valves and great vessels tolerably healthy.

*Abdomen.* Great omentum, which was loaded with fat, firmly adherent to the parietal peritoneum. Liver much enlarged, forming a tumor of some size extending into the left hypochondrium, and downwards, considerably below the umbilicus: its peritoneal covering adhered firmly to the parts with which it was in contact. The increased size of the liver depended principally upon enlargement of the left lobe.

On examining the opening which had been made during life, it was found to lead into a considerable cavity, situated on the upper surface of the right lobe of the liver near its free margin, bounded by dense and firm adhesions which had taken place between the peritoneum covering the liver, and that lining the parietes of the abdomen; the abscess, however, extended into the interior of the substance of the liver, by a large ulcerated opening, and had made its way also to the under surface of the viscus, forming a large collection of matter in the situation of the gall-bladder, no trace of which could be discovered, its place being entirely filled up by the pouch of matter. On tracing the gall-duct from the intestine, it was found to proceed direct to this cavity, from which it was merely separated by a barrier of lymph. The abscess had also made its way, by ulceration, into the cæcum, a large opening into which existed nearly opposite the ileo-cæcal valve.

Structure of the liver somewhat indurated. The right lobe presented a curious lobulated appearance, being divided into two or three smaller lobes. Kidneys healthy.

Other viscera not examined.

*Remarks.*—When first this officer was visited by Mr. Martin, he entertained some hopes of his recovery; and the result of treatment during several weeks warranted that view of the case. But the unfortunate and irregular plan pursued by the sufferer, especially in the use of mercury, tended to his injury by encreasing the general and local irritation which it could not, under the circumstances of the case, relieve. The subject was cachectic, and the local action appeared altogether chronic, until altered by the operation of mercury. The state of the constitution will account for the absence of many of the symptoms usually characterising hepatic abscess, such as rigor and sweat; and Mr. Martin is disposed to refer the recent untoward change in this unfortunate officer's condition entirely to the use of mercury and exposure to cold. When, on the 28th of September, he presented himself to Mr. Martin, there was death stamped in his very countenance. The destruction of the gall-bladder was a remarkable feature in the disease, and so was the implication of the cæcum—a condition remarked by Mr. Martin as of frequent occurrence in tropical dysentery, when complicated with hepatic disease.

### The Echometer.

A talented young physician, Dr. Aldis, has invented this instrument for the purposes of percussion, for which it seems well adapted. It may be procured at Savigny's. The percussor is attached to one end of a lever handle, which is fixed on the shaft of the instrument, like the lever in the Assalini's tenaculum. A screw under the lever handle regulates the depth to which it can be depressed, and consequently the height to which the percussor can be raised at the other end, as well as the force of the blow from the percussor. Equal sounds are consequently produced. The instrument is a very ingenious, and likely to prove a very useful one to auscultators.

---

